# Information dynamics in neuromorphic nanowire networks

**DOI:** 10.1038/s41598-021-92170-7

**Published:** 2021-06-22

**Authors:** Ruomin Zhu, Joel Hochstetter, Alon Loeffler, Adrian Diaz-Alvarez, Tomonobu Nakayama, Joseph T. Lizier, Zdenka Kuncic

**Affiliations:** 1 School of Physics, The University of Sydney, Sydney, NSW 2006 Australia; 2 International Center for Materials Nanoarchitectonics (WPI-MANA), National Institute for Materials Science (NIMS), 1-1 Namiki, Tsukuba, Ibaraki 305-0044 Japan; 3 Graduate School of Pure and Applied Sciences, University of Tsukuba, Tsukuba, Japan; 4 Centre for Complex Systems, Faculty of Engineering, The University of Sydney, Sydney, NSW 2006 Australia; 5 Sydney Nano Institute, The University of Sydney, Sydney, NSW 2006 Australia

**Keywords:** Information theory and computation, Electronic properties and materials, Nanowires, Network models, Electronic properties and materials, Nanowires, Complex networks, Nonlinear phenomena, Dynamical systems, Network models

## Abstract

Neuromorphic systems comprised of self-assembled nanowires exhibit a range of neural-like dynamics arising from the interplay of their synapse-like electrical junctions and their complex network topology. Additionally, various information processing tasks have been demonstrated with neuromorphic nanowire networks. Here, we investigate the dynamics of how these unique systems process information through information-theoretic metrics. In particular, Transfer Entropy (TE) and Active Information Storage (AIS) are employed to investigate dynamical information flow and short-term memory in nanowire networks. In addition to finding that the topologically central parts of networks contribute the most to the information flow, our results also reveal TE and AIS are maximized when the networks transitions from a quiescent to an active state. The performance of neuromorphic networks in memory and learning tasks is demonstrated to be dependent on their internal dynamical states as well as topological structure. Optimal performance is found when these networks are pre-initialised to the transition state where TE and AIS are maximal. Furthermore, an optimal range of information processing resources (i.e. connectivity density) is identified for performance. Overall, our results demonstrate information dynamics is a valuable tool to study and benchmark neuromorphic systems.

## Introduction

The brain is recognized as a powerful and efficient information processing system^1,2^. Its network structure has inspired a plethora of artificial neural network algorithms now widely used in machine learning^3,4^. Notwithstanding the success of these neuro-inspired learning approaches, the physical nature of real neurons and synapses has also inspired hardware-based approaches to replicating the unique information processing capabilities of biological nervous systems^5^. A key milestone in this endeavour was the dramatic reduction in power consumption achieved by integrating memory and processing in so-called neuromorphic chips^6^. This has been exploited to improve the efficiency of neural network training, a computationally intensive task. Other neuromorphic computing approaches have successfully demonstrated neuron spike-based learning implemented in CMOS hardware^7–9^ and synapse-based learning achieved using novel post-CMOS device components with resistive memory (memristive) switching properties^10–15^.

Neuromorphic information processing capabilities have also been demonstrated in low-dimensional nanomaterials constructed by bottom-up methods, e.g. quantum dots^16^, carbon nanotubes^17^, nanoparticles^18^ - see Sangwan and Hersham (2020)^19^ for a comprehensive review. These represent a unique class of neuromorphic systems, as bio-inspired self-assembly can produce highly disordered structures with emergent collective computational abilities^20,21^. This draws strong similarities to complex systems approaches used to study the brain’s network dynamics^22^. The present study focuses on neuromorphic Nanowire Networks (NWNs), comprised of metallic nanowires that self-assemble to form a complex network topology, with memristive cross-point junctions^23^. Previous studies have shown that NWNs exhibit complex, neuromorphic structural properties such as small-worldness, modularity and recurrent feedback loops^24^. In response to electrical stimulation, NWNs exhibit emergent collective neural-like dynamics^25–31^, including a “winner-takes-all” (WTA) electrical transport path^18,27–30,32,33^. This feature emerges above a threshold voltage when connected edge junctions collectively switch to a low resistance state, thereby activating the network into a highly conducting state. Theoretical studies suggest that a WTA gate module in recurrent neural networks may be optimal for computation^34^. Furthermore, activation of disordered memristive networks may be concomitant with a phase transition^35^, which is often associated with optimal information processing capabilities at the edge-of-chaos^36,37^. Simulation studies^38–41^ and experimental measurements^25,26^ using a multi-electrode array device have demonstrated neuro-inspired learning using a reservoir computing approach. In that approach, only the (memoryless) readout layer is trained, while the network dynamically self-adjusts its synapse-like memristive junctions in response to changing input signals.

This study aims to gain deeper insights into the neuromorphic information processing capabilities of NWNs by quantifying the dynamics of information transfer and storage. The information dynamics measures of transfer entropy (TE) and active information storage (AIS) have been used to quantify the intrinsic nonlinear dynamical properties of complex systems, especially systems performing distributed computing^42–48^. Whilst mutual information has previously been applied to neural network algorithms^49,50^, this metric does not capture the dynamical properties of directed information flow and storage. In contrast, TE quantifies how much information the past activity of a source component provides about the state update of a target component (beyond that provided by the target’s own past) and captures directed information flow between the components^42,48^. AIS quantifies how much information the past activity of a component provides about its next value, capturing the memory that is active in the computation to update that component’s state^45^. TE is particularly useful in measuring how information carried by signals is propagated across a network, and can help identify the most important components involved in the dynamics of a network^44,51^, and performance of the network can thus be optimized accordingly^52^.

Crucially, TE and AIS have been used to study the dynamics of intrinsic information processing in many other systems undergoing order-chaos phase transitions through critical-like points, including canonical complex systems such as Random Boolean Networks^53,54^ and the Ising model^55–57^, echo state networks^58^, spiking neural networks^59^, in vitro neural networks^60^, and neural mass models^61^. Typically, TE and AIS indicate maximisation of information storage and transfer in the intrinsic information processing at or near the edge-of-chaos, and also often point to a balance between these operations in this regime. Such a maximisation of information processing capabilities in intrinsic dynamics provides an explanation for optimal computational capabilities found at the edge-of-chaos in some systems such as recurrent neural networks^36,58^ (and conjectured in others). In a related study to ours, Cramer et al.^62^ measured TE and AIS in a different neuromorphic system, finding that information transfer and storage in the intrinsic dynamics of the system increased as the system approached criticality from an asynchronous/bursting state. Whether and how those findings extend to neuromorphic NWNs—where nodes undergo inactive-active transitions not only individually but strongly in tandem with their ongoing time-series activity rather than in response to some external tuning—and how this relates to implementations of reservoir computing therein, is an open question.

This work is organized as follows. First, we present the spatio-temporal correlation between properties of the NWN components and their information dynamics. Then we show the time-series evolution of relevant network properties, including average TE and AIS. We then present results showing how a network’s performance in learning and memory can be interpreted in terms of the information dynamics measures. We also present results showing the influence of network connectivity density on information processing performance.

## Results

Here, we use simulations based on a physically-motivated model^30,39,40,63^ to identify the junctions responsible for signal transmission and investigate the corresponding dynamical network characteristics in terms of the information theoretic measures TE and AIS. The model captures the structural features of nanowire network self assembly. Nanowire-nanowire intersections are modelled as memristive electrical junctions, based on an experimental system^30^. An externally applied voltage is redistributed across the networked junctions (in accordance with Kirchoff’s circuit laws), causing some to switch from a low-conductance state to a high-conductance state (see “Methods” for details). Time-series analysis and benchmark learning and memory tasks are used to demonstrate how TE and AIS can provide new insight into the dynamical information processing capacity of NWNs.

### Junction centrality and information flow

#### Junction state and network current flow

In network analysis, centrality is a key metric that can help identify the relative importance of nodes in relation to the underlying network structure and connectivity^64,65^. Despite the existence of numerous classes of centrality algorithms, betweenness centrality can be considered as one of the most well-known measures as it quantifies how often a node (or edge) performs as a “bridge” on the shortest paths between arbitrary nodes in the network^66,67^. This study employs a variation of betweenness centrality based on current-flow^68^, relevant for nodes and edges within electrical systems (see “Methods” for details). At a specific time *t* during network activation, current-flow edge betweenness centrality $c_{\scriptscriptstyle {\mathrm EB}}$ is calculated using Eq. (7).

**Figure 1:**
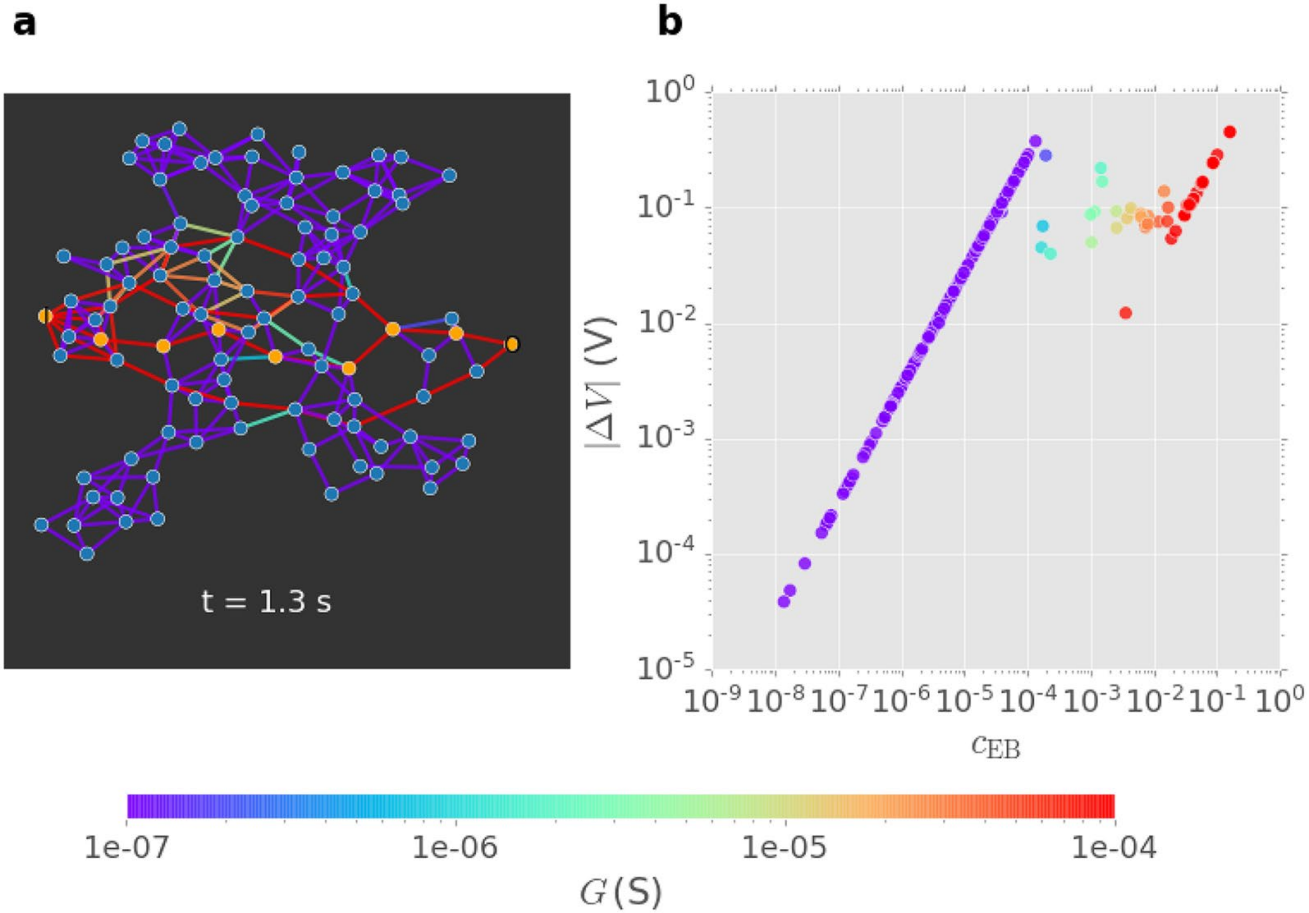
Simulation of a neuromorphic NWN with 100-nanowire nodes and 261-junction edges. (**a**) Snapshot of network graph at $t = 1.3\,$s, showing the first current path (between orange nodes, established earlier at $t = 1.218$ s) and edge (junction) conductance (colourbar). (**b**) Magnitude of voltage difference $|\Delta V|$ and conductance *G* across network junctions as a function of their current edge betweenness centrality $c_{\scriptscriptstyle {\mathrm EB}}$ at $t = 1.3$ s.

Figure 1a shows a snapshot of the network graph at $t = 1.3$ s. The orange nodes indicate the first current transport path, formed earlier at $t = 1.218$ s and coinciding with network activation. This WTA path is equivalent here to the shortest path length between the source and drain nodes. The locations of high-conductance junctions beside the WTA pathway demonstrate the importance of centrality—junctions at more central locations typically switch on earlier and thus regulate the subsequent dynamics of information spread by current. This suggests that the interplay between network connectivity and junction dynamics results in adaptive information flow.

Figure 1b plots the magnitude of voltage difference $|\Delta V|$ across all network junctions as a function of $c_{\scriptscriptstyle {\mathrm EB}}$ at $t = 1.3$ s, with colour indicating each junction’s conductance state. Three regimes can be clearly identified: (i) an extensive linear regime spanning approximately 4 orders of magnitude in both $|\Delta V|$ and $c_{\scriptscriptstyle {\mathrm EB}}$ characterized by low conductance ($\lesssim 10^{-6}$ S) states; (ii) a shorter transition regime with intermediate conductance values ($10^{-4} - 10^{-5}$ S), corresponding to junctions in the tunneling transport regime; and (iii) a short linear regime characterized by high conductance ($\gtrsim 10^{-5}$ S) states, corresponding to junctions that have switched on and carry Ohmic current. Junctions in the high conductance regime therefore form the WTA path, pushing the collective conductance of the network to a higher level. Furthermore, junctions in the transition regime will form subsequent current paths. Within the low conductance regime, junctions with higher $c_{\scriptscriptstyle {\mathrm EB}}$ enter the tunneling regime earlier and thus switch on earlier. For instance, at $t=0$ s, all junctions reside in the low conductance regime. The centrality $c_{\scriptscriptstyle {\mathrm EB}}$ at $t=0\,$s reflects the order of junctions to be turned on through the activation process. These results show that junctions in the highest conductance state are generally also those with the highest centrality.

#### Transfer entropy: information flow

The adaptive dynamics of NWNs makes them ideally suited to temporal information processing tasks^27,30^. In general, the performance of a given network structure depends on the specific task^38–41,69,70^. However, information theory provides an effective approach to assessing and characterising intrinsic network performance in a task-independent manner^71,72^. Information theoretic measures enable the quantification of the information transfer between nodes and associated information storage^43–45,47,73^. Transfer entropy (TE), in particular, is a key information theoretic measure of predictive information provided by a source node to a destination node^43,44^. Estimation of TE enables identification of information propagation and thereby active areas of a given network structure. In this study, TE is based on edge connectivity—for each edge (junction) in the network, TE is estimated in both directions and the sum measures net activity on the edge. For the following results, 50 network simulations were performed with different source-drain locations (keeping the graphical distance between these nodes constant).

**Figure 2:**
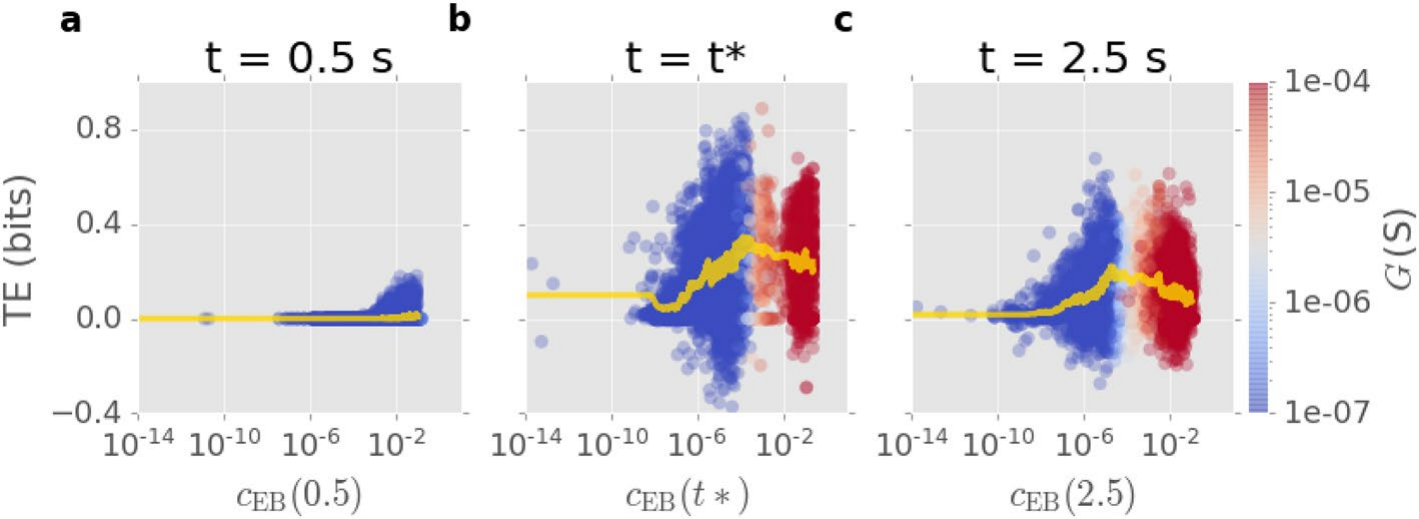
Junction TE as a function of edge betweeness centrality $c_{\scriptscriptstyle {\mathrm EB}}$ calculated for 50 simulations of a 100-node network with varying source-drain locations (fixed distance) at three different time points: (**a**) $t = 0.5\,$s; (**b**) $t = t^*$, where $t^*=1.22$ s is network activation time; and (c) $t = 2.5\,$s. Each data point represents one junction at the specified time. The colourbar represents the corresponding junction conductance. The yellow curve is a moving window average (size 0.1 s) of the scatter points calculated by sorting the scatter points based on their $c_{\scriptscriptstyle {\mathrm EB}}$ values and averaging $c_{\scriptscriptstyle {\mathrm EB}}$, TE values within each window.

Figure 2 shows junction TE as a function of $c_{\scriptscriptstyle {\mathrm EB}}$ at $t = 0.5$ s, $t = t* = 1.22$ s, and $t = 2.5$ s, where $t*$ represents the network activation time. Each TE-$c_{\scriptscriptstyle {\mathrm EB}}$ data point on the scatter plot represents one junction in 50 network simulations. Since TE estimation at a specific time point includes bias due to the estimator (see “Methods”), a window of 100 time steps ($0.1 \,$s) is employed to mitigate uncertainty. Figure 2a shows TE vs $c_{\scriptscriptstyle {\mathrm EB}}$ at $t = 0.5$ s. At this time, networks are in a pre-activation stage. Most junctions remain in a low conducatance state. TE for most junctions is negligible. Only junctions with the highest $c_{\scriptscriptstyle {\mathrm EB}}$ values exhibit small TE fluctuations.

Figure 2b shows TE versus $c_{\scriptscriptstyle {\mathrm EB}}$ at $t = t*$. At this time, the most central junctions ($c_{\scriptscriptstyle {\mathrm EB}}\gtrsim 10^{-2}$) switch on (red), while less central junctions (blue) exhibit higher TE and thus stronger information dynamics. A notable number of junctions are in the transition regime, between off and on ($10^{-4} \lesssim c_{\scriptscriptstyle {\mathrm EB}}\lesssim 10^{-2}$). The moving window average suggests that junctions entering this regime ($c_{\scriptscriptstyle {\mathrm EB}}\sim 10^{-4}$) have the highest TE values and are thus poised to transmit substantial information.

Figure 2c shows TE versus $c_{\scriptscriptstyle {\mathrm EB}}$ at $t = 2.5 \, s$, at which point networks have activated. Here, fewer junctions are in the transition regime compared to $t = t*$, as most are already switched on. Again, more central junctions exhibit stronger information dynamics, and junctions entering the transition regime have the highest TE. However, TE at this network state is generally lower compared to at $t = t*$. Information dynamics thus tends to be weaker after activation, as fewer junctions are in the process of switching.

These results demonstrate that the structure and connectivity of the network plays an important role in the information dynamics of neuromorphic NWNs. Junctions at more central positions tend to exhibit stronger TE. Similar results can be observed for AIS as well (see supplementary Fig. 1). Collectively, information transfer and storage achieves a higher level when the network begins to activate. Locally, at the junction level, network activation coincides with junctions switching to a high conductance state.

### Network dynamics of information transfer and storage

#### Network time series

**Figure 3:**
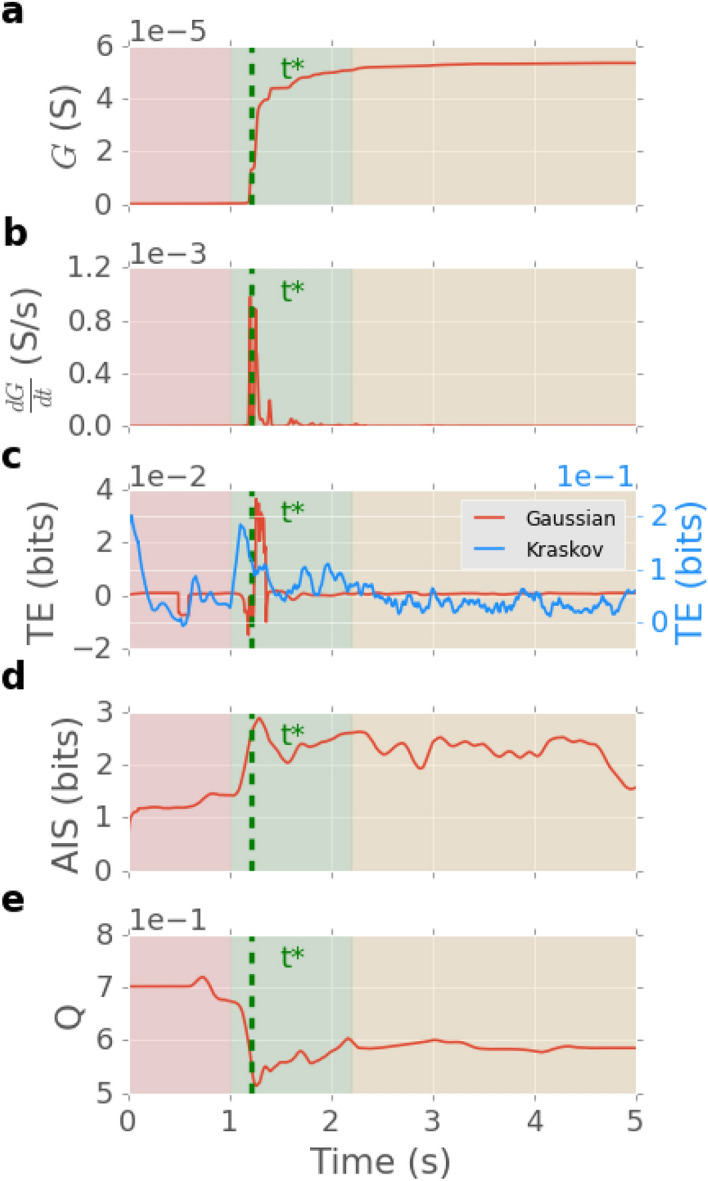
Network time series. (**a**) Network conductance *G*. (**b**) Time derivative of *G*. (**c**) Transfer entropy TE, calculated with the Gaussian and Kraskov estimators and averaged over all junctions. (**d**) Active information storage AIS. (**e**) Network modularity *Q*. Red, green and beige shaded regions indicate pre-activation, activation and post-activation periods, respectively, for a Mackey-Glass input signal. The dashed vertical green line indicates the activation time, $t^* =1.22\,$s, coinciding with formation of the first current path.

Figure 3 shows the time-series of key network properties in response to a Mackey-Glass input signal (with $\tau = 50$): (a) conductance *G*; (b) its derivative $\delta G/\delta t$; (c) TE, calculated for both Gaussian and Kraskov estimators, and averaged over all junctions; (d) AIS (Kraskov); and (e) conductance-weighted modularity *Q*, which is a metric that captures and characterizes the community structure of networks (see “Methods”)^74–76^. The time series is split into three regimes: pre-activation, activation and post-activation, shaded red, green and beige, respectively.

In the pre-activation regime, all junctions are off, with very low *G*. Junction conductance states start to evolve based on their voltage. *G* increases initially in junctions with higher voltages. TE increases when the signal is first applied to the network, but in general remains relatively low in this regime. AIS remains relatively low in this regime since there is little memory of past states. Here only AIS calculated based on the Kraskov estimator is presented, since little linear information storage was captured here by the Gaussian estimator. *Q* remains constant since all junctions remain in a low-*G* state. The activation regime is characterised by a steep increase in *G*, sharp spikes in $\delta G/\delta t$, a peak in TE and AIS, and a drop in *Q*. During activation, several neigbouring central junctions switch on and *G* jumps, resulting in time derivative spikes. The first spike in $\delta G/\delta t$ coincides with the formation of the first WTA current path at $t = t^* =1.22$ s (green dashed line). This also coincides with a sharp dip in *Q*, which indicates that the network suddenly becomes highly integrated. TE also exhibits dramatic changes near $t^*$, particularly for the Gaussian estimator. The Kraskov-estimated TE shows the largest increase just before $t^*$, which may suggest potential predictive capacity. Indeed, the TE results from this non-linear estimator were an order of magnitude larger than those of the Gaussian estimator near the activation time, suggesting strong non-linear interactions in that regime. In the post-activation regime, many junctions are in high-conductance states and consequently, *G* is in a high stable state. TE returns to a low, approximately constant level, indicating little information flow. In contrast, the AIS increases to a higher level at activation and remains there post-activation; in conjunction with the lower information flow this indicates more rich, self-predictable dynamics of the nodes in comparison to pre-activation. *Q* remains stable in this regime and at a lower level than during pre-activation, indicating the network has evolved to a more integrated state post-activation.

**Figure 4:**
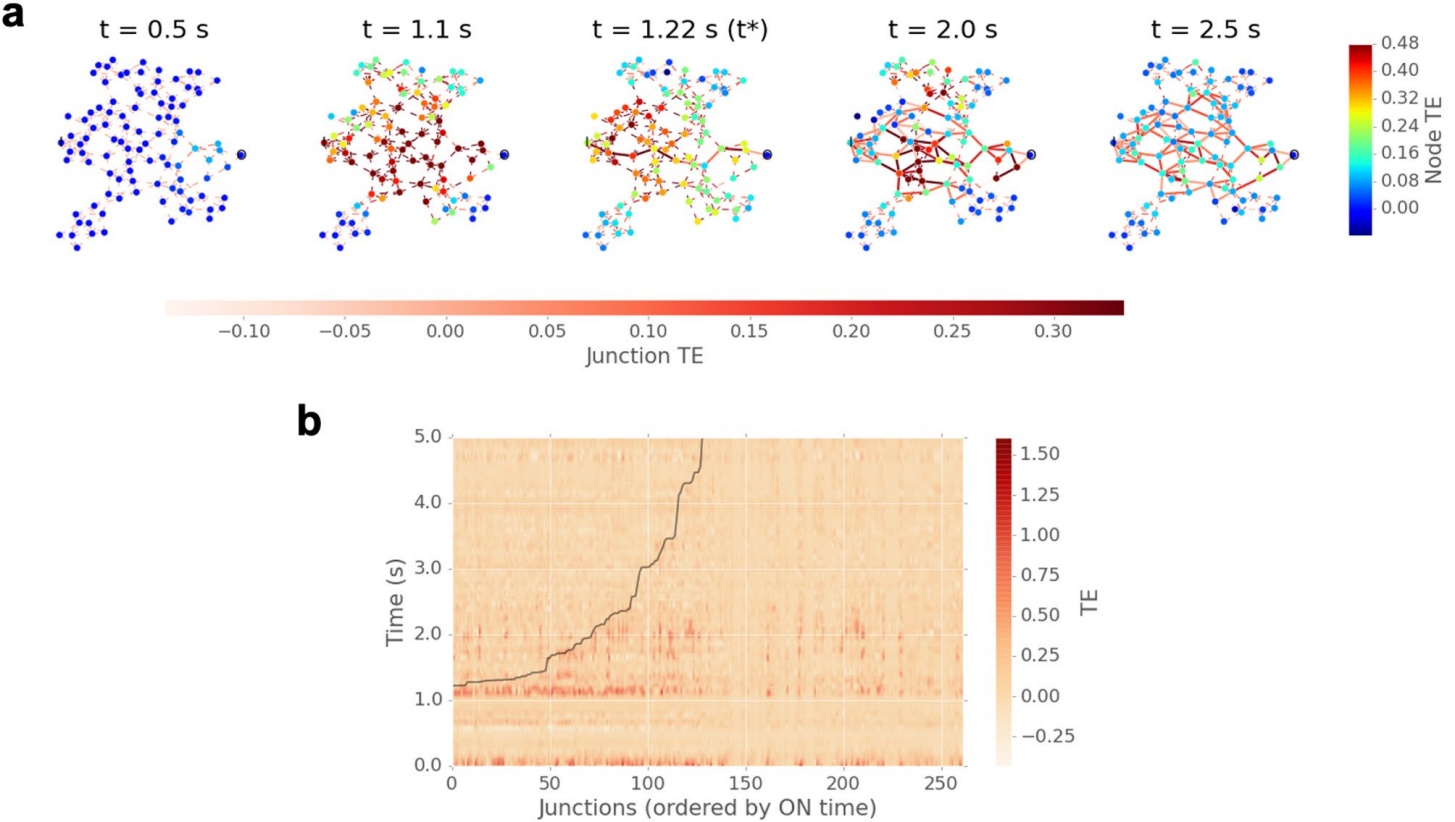
Network information flow dynamics. Top panel—Snapshots of the network taken at time points before activation ($t = 0.5$ s), at the onset of activation ($t=1.1\,$s), when the first current path forms ($t=1.22$ s), and after network activation ($t = 2.0, 2.5$ s). Nodes are colored with time-averaged TE flow (inTE + outTE) within the last 0.2 second window using the Kraskov estimator. Junctions are colored by the sum of corresponding TE in both directions. Bottom panel—heatmap of junction TE as a function of time, with junction number ordered by switch on time.

Snapshots of the network at different stages of activation are shown in Fig. 4a. Here, nodes and edges are colored by their respective TE (Kraskov) averaged via a moving time window (0.1 s). Several time points are chosen from the three regimes referred to in Fig. 3. At $$t = 0.5 $$ s, the whole network is in the pre-activation regime, during which information dynamics are barely discernable. At $$t = 1.1 $$ s, when the network is approaching activation, the majority of nodes/edges are involved in the information dynamics. The most active nodes and edges are observable around the center of the network (i.e. high $$c_{\scriptscriptstyle {\mathrm EB}}$$), between source and drain. At $$t = t^* = 1.22$$ s, the WTA path emerges from the area that was previously most active (at $$t = 1.1 $$ s), while areas adjacent to the WTA path also exhibit strong dynamics. These areas form additional current pathways at later times, as evident at $$t = 2.0 $$ s. At this point, the network’s activation winds down and its dynamics start to fade. At $$t = 2.5 $$ s, the network is in the post-activation regime, where—despite the conductance being maximal—the information dynamics decrease to a lower (but still observable) level. These network snapshots also highlight the predictive capacity of TE activity. Network nodes/edges with the strongest TE dynamics (e.g. at $$t = 1.1 $$ s) are subsequently involved in the first current path formation and ongoing network activity.

Figure 4b shows a heat map of each junction’s TE during activation. Here the *x*-axis represents junction indices ordered by the time they switch on (i.e. lower means earlier). The black curve denotes their corresponding switch on time. Hot zones (dark red) in the heat map demonstrate strong transfer events occur just before junctions switch on, particularly for those that switch on earlier and are involved in network activation. Based on the results from Fig. 2b, the more central a junction is, the earlier it switches on and the stronger information dynamics it exhibits. Weaker TE events that are still evident at later times correspond to junctions whose conductance continues to evolve, but does not reach a sufficiently high state for the junction to switch on. Some TE events evident on the right half of the panel are not coupled with junction switching behaviors. These dynamics are induced by the collective conductance change of the network due to the WTA path formation.

### Memory capacity and learning

The previous results shown in Fig. 4 reveal how information is dynamically transmitted through a neuromorphic NWN. Here, we investigate information storage and transfer dynamics involved in two different reservoir computing benchmark tasks: memory capacity (MC)^77^ and non-linear transformation (NLT)^38^.

#### Network pre-initialisation

As shown in Figs. 3 and 4, the continuously evolving internal states of a NWN give rise to network dynamics with distinctive features around activation. Thus, we pre-initialised networks with different initial states (i.e. pre-activation, activation and post-activation states) to investigate the influence of initial network state on information storage and transfer, as measured by AIS and TE, respectively. Networks were pre-initialised using the same Mackey-Glass signal as for Fig. 3. Network states, defined as the instantaneous junction conductance state distribution, were recorded every 50 time steps (0.05 s) and used as the initial states for the MC and NLT tasks. The time at which the network state was sampled is defined as its pre-initialization time. A pre-initialisation time of zero corresponds to all junctions set to the same low-conductance initial state, as used in the results presented thus far.

**Figure 5:**
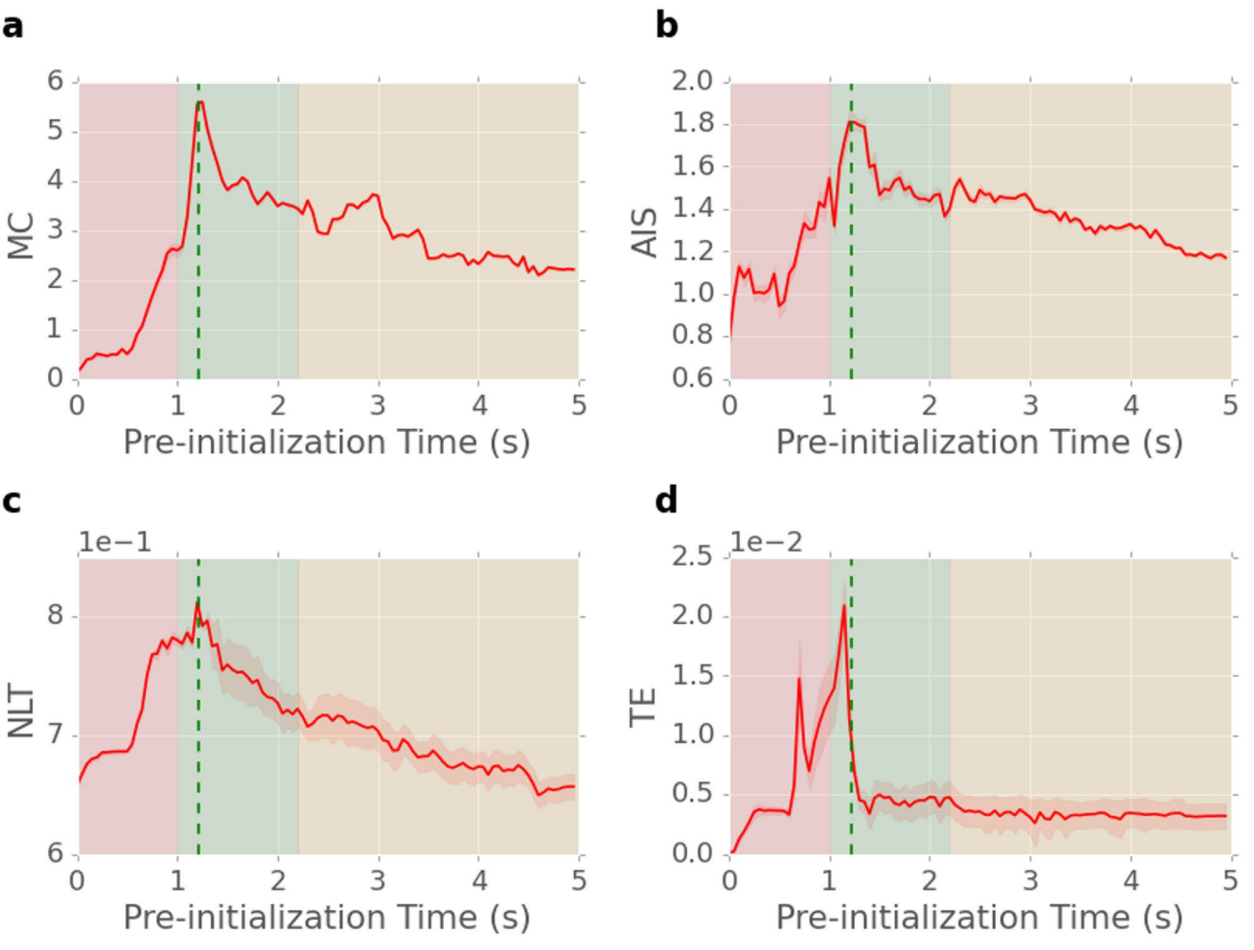
Information processing tasks and corresponding information dynamics measures for networks pre-initialised with different initial states (shaded regions represent standard error): (**a**) memory capacity (MC) task performance; (**b**) average active information storage (AIS) for MC; (**c**) non-linear transformation (NLT) accuracy; (**d**) average transfer entropy (TE) of NLT. Plots in (**a**) and (**b**) are averaged over multiple iterations of MC due to the inherent randomness of the task. Standard error is too small to be noticed. Plots in (**c**) and (**d**) are averaged over different amplitudes of the NLT task ranging between 0.6–1 V.

Figure 5a shows MC as a function of network pre-initialisation time. The corresponding AIS, averaged over its time-series, is shown in Fig. 5b. Both MC and AIS peak when the network is pre-initialised to the state at its activation time (green dashed line). Similarly, Fig. 5c shows NLT performance is optimal for pre-initialisation states approaching and at network activation. The corresponding TE, averaged over its time-series, as shown in Fig. 5d, also peaks when the network is pre-initialised to states approaching activation. These results reveal that network states at or approaching activation are optimal for these information processing tasks.

Information storage and transfer in the networks exhibits similar optimal behavior when the initial state approaches the critical-like network activation point. However, each task benefits from different network dynamical features. The MC task exploits the short-term memory of memristive elements in the network, as well as its recurrent structure. Information storage captures self-predictability at each node, incorporating strength of recurrence on nodes (i.e. relevant past information), and thus reflects the memory capacity of the network. The NLT learning task, on the other hand, involves the transfer and modification of a continuous signal and thus, the node-to-node predictive information contributing to the computation is best captured by TE. Furthermore, the energy-based metric “deviation from linearity”^78^ (see supplementary Fig. 2), which provides a measure of a network’s capability of nonlinearly transforming the input into different frequency components, is maximised when the network’s initial state approaches activation, which is optimal for both tasks.

#### Information processing capacity

As shown in the previous section, pre-initialisation enables optimal memory and learning performance. Here we investigate this further by considering networks of varying information processing capacity, as represented by average degree $\langle k \rangle$ (i.e. average number of connections per node). We generated 16 100-node NWNs with varying number of junctions (ranging from 261 to 4684), to determine whether increasing connectivity and therefore, information processing resources, increases network performance. We used the same MC task as in Fig. 5 to assess relative performance for different $\langle k \rangle$.

**Figure 6:**
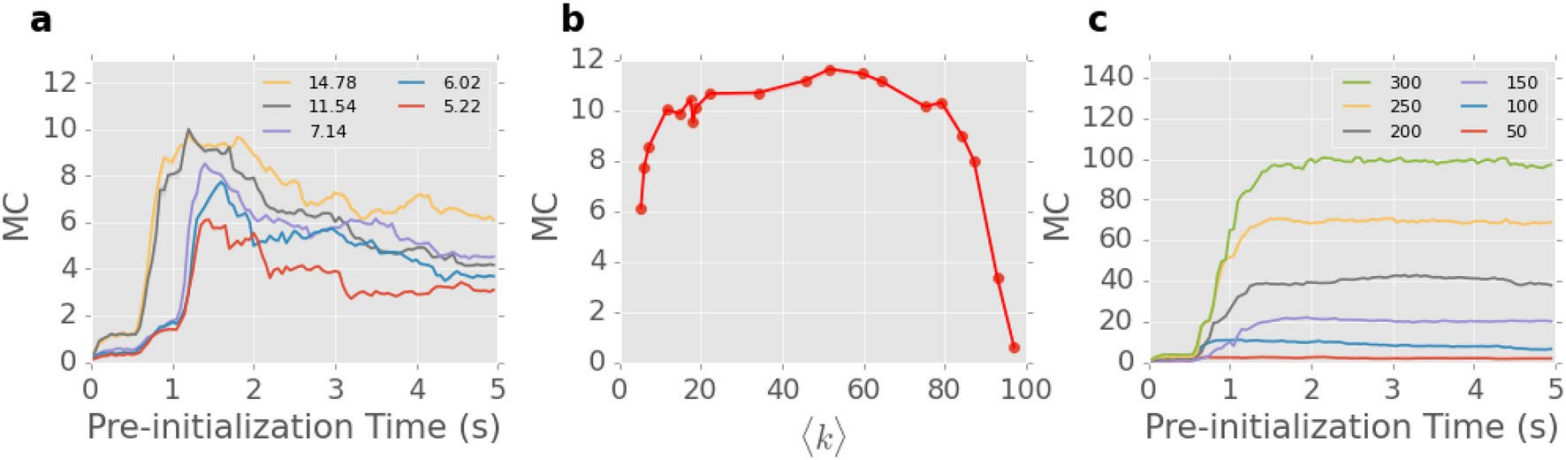
Memory capacity (MC) for networks with varying information processing resources. (**a**) Average MC as a function of pre-initialisation time for 100-node networks with sparse connectivity ($5.22 \le \langle k \rangle \le 14.78$). (**b**) Maximum MC achieved for 100-node networks as a function of $\langle k \rangle$. (**c**) Average MC vs. pre-initialisation time for networks with different number of nodes (50–300) and similar $\langle k \rangle$.

Figure 6a shows MC with respect to pre-initialization time for networks with relatively low $\langle k \rangle$ (5–15). Similar to the results in Fig. 5, MC is optimized when the networks are pre-initialised to their respective critical-like activation state. Additionally, networks with higher $\langle k \rangle$ tend to show better performance in the MC task when in this state.

Figure 6b shows the best MC performance of each of the 16 networks (pre-initialised to its optimal state) as a function of $\langle k \rangle$. Networks with $\langle k \rangle < 20$, show increasing performance as density increases, due to more possible current pathways being formed during network activation. For networks with $20<\langle k \rangle < 70$, performance plateaus, while for $\langle k \rangle > 70$, performance decreases significantly. These results indicate that there exist an optimal connectivity density for NWNs to perform information processing tasks. A small drop in MC can be observed for a specific network realisation with $\langle k \rangle \sim 20$, suggesting that other topological properties could affect the network’s memory as well. The optimal computational density regime coincides with the maximum deviation from linearity (see supplementary Fig. 2)—networks with better performance exhibit stronger deviation from linearity.

Figure 6c plots MC for networks of different sizes (number of nanowire nodes) but similar $\langle k \rangle (\approx 20)$, as a function of pre-initialisation time. A trend similar to Fig. 6a is evident—the best performance can be achieved with appropriate pre-initialisation. Furthermore, the plot demonstrates that larger networks tend to exhibit better performance as they are able to incorporate more information processing resources.

These results demonstrate that the optimisation of NWN memory and learning performance around critical-like points holds for different network realisations. For networks with the same number of nanowire nodes, there exists an optimal regime of $\langle k \rangle$ for the NWNs to exhibit the best performance. On the other hand, larger networks (more nanowires) generally makes the performance even better.

## Discussion

This study is the first to investigate the spatio-temporal dynamics of information transfer and storage in neuromorphic nanowire networks. We found that topological network dynamics, as measured by edge betweeness centrality $c_{\scriptscriptstyle {\mathrm EB}}$, influences information transfer, with more central junctions exhibiting stronger TE. We also found TE is maximised when individual junctions approach their respective transient dynamics regime (corresponding to a tunneling electron transport regime). These results corroborate those of Joyce et al.’s study, which showed that high centrality components in brain networks tend to be more influential in network dynamics^79^. However, the centrality metric used in that study was based on static structural properties and the effect on information dynamics was not investigated. TE in contrast has been widely used in neuroscience to study the dynamic activity in a network in terms of measuring information flow between nodes. This has included, for example, inferring functional/effective connectivity from analysing EEG/MEG data^44^ and spiking cortical networks^51^, as well as finding that TE is correlated to connectivity strength^80^.

Our time-series analysis (Fig. 3) revealed that during network activation, TE and AIS are maximized when the first (WTA) current path is about to form. Together with our previous results showing that neuromorphic nanowire networks exhibit ordered and chaotic dynamical regimes, as well as a regime near activation resembling a “critical-like” state at the edge-of-chaos under self-regulated dynamics^41^, these results corroborate several other analyses of information dynamics in systems undergoing order-chaos phase transitions through critical-like points such as random Boolean networks^53,54^, the Ising model^55–57^, echo state networks^58^ and other systems^59–61^. Some of these studies suggested maximisation of both information storage and transfer at the edge of chaos (e.g. in echo state networks^58^), whilst others found AIS peaks under ordered dynamics and TE peaks under chaotic dynamics with a balance between these operations at the edge of chaos (e.g. in random Boolean networks^53,54^). Our results align with the former group, with both TE and AIS peaking at the critical-like activation point. These differences may be attributed to the different types of networks and activation methods used in the different studies. Nevertheless, in both groups the edge of chaos represents a regime where both operations of information storage and transfer are strong, and the similarity of information dynamics behavior is intriguing, especially considering the suggestion that information processing may be optimised around the edge-of-chaos^34^. Additionally in our study, a local measure of TE on junctions exhibited a pre-emptive signature with respect to their individual switching behavior, echoing early information transfer observed prior to the critical point in the Ising model^55–57^ and in Kuramoto oscillator synchronisation^81^.

Neuroscience studies on network modularity typically investigate the relationship between a static modular structure (unweighted) and functionality^82,83^. Bassett et al. however, found that brain networks exhibit dynamic reconfiguration during learning and the modular community structure of the network changes with time^84^. In our study, we calculate modularity based on the evolving junction conductance. The corresponding time-series, Fig. 3e, indicates that modularity first increases when conductance increases across several junctions. The network then becomes most integrated (modularity minimized) at the critical-like point. This can be attributed to inter-module connections formed by stronger junctions and some nodes involved in the WTA path divested to form fewer larger modules (see supplementary Fig. 3). In a related study, Husken et al. investigated the evolution of modularity in feed-forward neural networks in a task-dependent manner^85^. They found modularity increases as the learning process continues before dropping to a steady-state. A similar peak in modularity evolution with respect to training was also shown by Shine et al. in deep neural networks performing the MNIST digit recognition task^86^. They found that learning accuracy saturates at maximum modularity. Our results likely reflect the small-world and recurrent structure of NWNs, which are fundamentally different from feed-forward artificial neural networks.

Our study also revealed that learning performance (NLT) and memory capacity (MC) of NWNs can be optimized by pre-initialising the network to its critical-like activation state, where AIS and TE are both optimized (Fig. 5). A similar approach was also employed by Klos et al. to perform dynamical learning of different tasks. They found that appropriate pre-initialisation enables the network to better adapt to a target^87^. Our results are also consistent with those of Boedecker et al.’s investigation of optimal network performance around the edge-of-chaos in RBNs^58^ and Bertschinger et al.’s results using recurrent neural networks^36^. Furthermore, Cramer et al.’s study on criticality in spiking neural networks implemented on a memristor hardware system showed that tasks of different computational complexity benefit by varying amounts from the network’s dynamical regimes^62^. In our study, the MC task, which measures short-term memory, correlates with AIS dynamics, while the NLT task correlates with TE dynamics. Another metric called ’deviation from linearity’ has been proposed to measure non-linearity within reservoir systems^78^. Butcher et al. found that larger deviation from linearity correlates with better performance for a reservoir with random static projections (${\mathrm {R^2SP}}$)^88^. Similarly, we found that the best memory and learning performance of pre-initialised NWNs correlates with the maximum deviation from linearity based on the network’s intrinsic dynamics. We also found that the learning performance of pre-initialised NWNs correlates with “flexibility” (see supplementary Fig. 4), measured by the frequency of changes in network modular structure, as proposed by Bassett et al.^84^.

The performance of conventional silicon based chips is mainly determined by the number of transistors, which has doubled every two years in accordance with Moore’s law. In the context of post-Moore’s law information processing with neuromorphic reservoir networks, studies have shown that the performance can be strongly affected by the network size, structure and connectivity^89^. Our results (Fig. 6) show that, unlike traditional CMOS chips, the performance of NWNs does not increase monotonically with respect to its connectivity, i.e. computational density. Instead, there exists an optimal range of $\langle k \rangle$. In addition, larger networks tend to exhibit significantly better performance when pre-initialised. These results agree with Legenstein et al.’s results on the computational performance of neural circuit models, which suggest that an optimal $\langle k \rangle$ may be beneficial for computing, especially at the edge-of-chaos^37,90^. Our finding of an optimal range of $\langle k \rangle$ for information processing also corroborates similar findings by Snyder et al. in their studies on RBNs and random automata networks^91,92^. Furthermore, we found that networks within the optimal range of $\langle k \rangle$ exhibit larger deviations from linearity (see supplementary Fig. 2).

All the simulations presented here, including network pre-initialisation, were performed using one input node. However, more complex tasks would generally require input signals to be delivered to multiple input nodes^40,93^, which in general would change the pre-initialised network states. In a real experimental hardware device, it would be difficult to control pre-initialisation to an optimal state. However, other studies suggest that some pre-initialisation, even if not to optimal states, could still be beneficial to learning tasks, enabling strategies such as transfer learning^41^.

In conclusion, we investigated the spatio-temporal dynamics of information transfer and storage in neuromorphic nanowire networks subject to a complex time-varying electrical signal. Our results demonstrate that information dynamics metrics provide a valuable tool for analysing the information processing capability of neuromorphic systems. We found information dynamics to be maximised around the critical-like activation point in nanowire networks, which agrees with previous studies on other similar computing systems based on recurrent neuromorphic networks. Futhermore, based on the information dynamics, we found that pre-initialisation enables these networks to achieve optimal performance in memory and learning tasks. We also found that there exists an optimal range of network density for computation. These results warrant further studies of information dynamics involved in more computationally complex tasks that are non-static, such as action recognition.

## Methods

### Electrical network model

**Figure 7:**
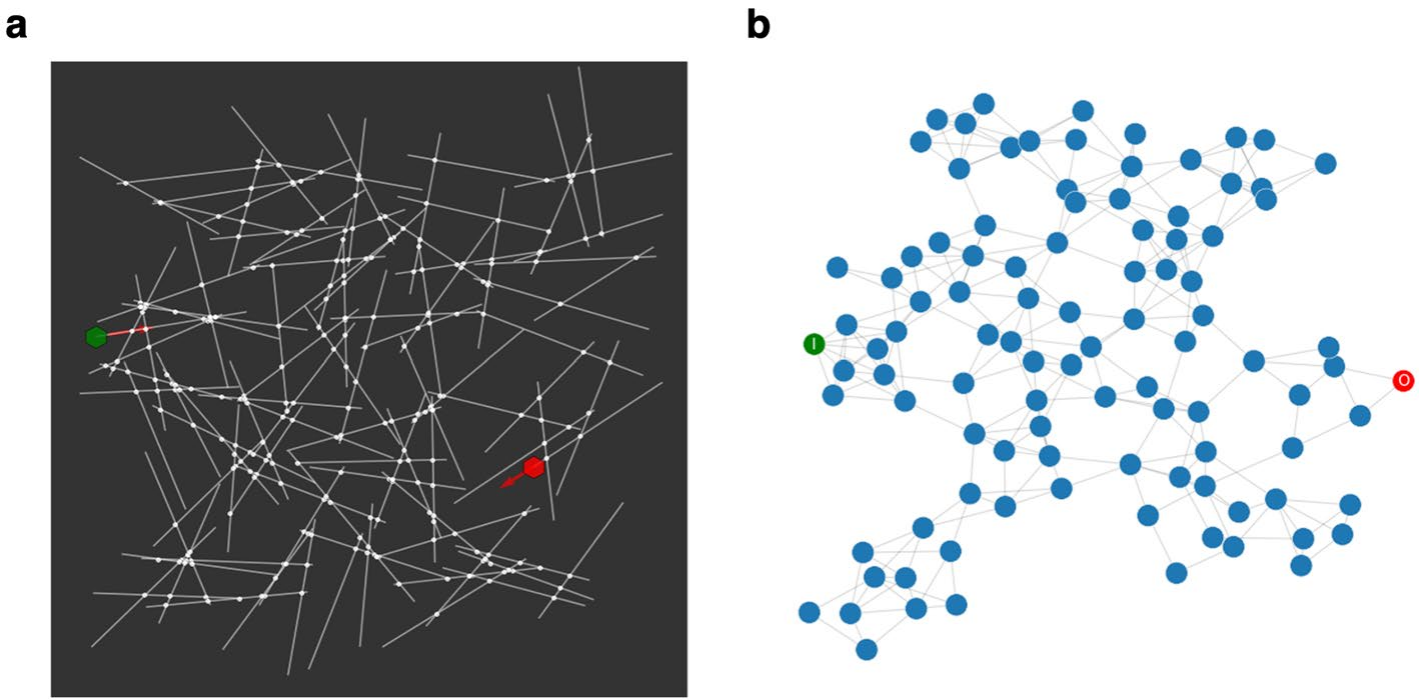
(**a**) Simulated NWN with 100 nanowires and 261 junctions. (**b**) Graphical representation of the NWN in (**a**). Green denotes input nanowire contact electrode-node and red represents drain node.

Figure 7a shows a visualisation of a simulated nanowire network containing 100 nanowires and 261 junctions. Self-assembly is modelled by distributing nanowires on a $3 \times 3 \,\mu {\mathrm{m}}^2$ 2D plane, with their centers uniformly sampled from [0, 3] and orientation uniformly sampled from $[0, \pi ]$. The lengths of the nanowires are sampled from a gamma distribution (mean = $100 \,$nm, standard deviation $10 \,$nm), based on experimental measurements^30^. Figure 7b shows the graphical representation of the NWN in Fig. 7a. Nanowires are represented as nodes, while junctions are represented as edges between corresponding nodes.

Unless otherwise specified, all simulation results are obtained using a NWN with 100 nanowires and 261 junctions. All variables, except the adjacency matrix *A*, are time-dependent. Two nodes on the left and right end of the network (as shown in Fig. 7) are used as source and drain electrodes. A Mackey-Glass time-series signal with delay parameter $\tau = 50$ is used as the input voltage signal, delivered to the source electrode. Kirchhoff’s voltage and current conservation laws are solved at each time step using the nodal analysis approach applied to the graphical representation of the network^94^. The junction conductance distribution evolves as voltage bias is continuously applied to the network. The Laplacian matrix of the junction conductance is used to describe the network’s state. Hence, at each time step, the junction voltage distribution *V* across the network is obtained by solving^95^

1 $$\begin{aligned} {{\mathcal {L}}}^\dagger V = I \qquad , \end{aligned}$$

where *I* is current and ${\mathcal {L}}^\dagger$ is the expanded graph Laplacian of the network, expressed in block matrix representation as

2 $$\begin{aligned} {\mathcal {L}}^\dagger = \left[ \begin{array}{c|c} {\mathcal {L}} &{} C\\ \hline C &{} 0\\ \end{array} \right] \qquad , \end{aligned}$$

where ${\mathcal {L}}$ is the graph Laplacian and where the elements of *C* are either 1 if the nanowire node is connected to an external electrode or 0 otherwise. The Laplacian is

3 $$\begin{aligned} {{\mathcal {L}}} = D - W \qquad , \end{aligned}$$

where *W* is the weighted adjacency matrix of the network, with the weights representing junction conductance distribution:

4 $$\begin{aligned} W_{ij} = A_{ij} G(i,j) \qquad , \end{aligned}$$

where *G*(*i*, *j*) is conductance on the edge connecting nodes *i* and *j* and *D* is the weighted degree matrix generated from *W*:

5 $$\begin{aligned} D = {\mathbf{diag}} (d_i) \qquad , \qquad d_i = \sum \limits _{k=1}^{N} W_{ik} \qquad . \end{aligned}$$

### Junction model

Nanowire-nanowire cross-points are modelled as ideal, electrically insulating, ionically conducting junctions, with voltage-threshold memristive switching^30,38,39,96^, modulated by electron tunnelling transport^97^. The model does not take into account noise fluctuations, which can contribute additional nonlinearities that can generate even richer dynamics^97^. Junction conductance, $G = G(\lambda )$, depends on a state variable $\lambda (t)$ that parametrises the conducting filament responsible for memristive switching. All junctions are initially in a high resistance “off” state. For each junction in the network, resistance switches to “on” state when $\lambda \ge \lambda _{\text {crit}}$, where $\lambda _{\text {crit}}$ is a set threshold. The ratio of these resistance states is $R_{\text {off}}/ R_{\text {on}} = 10^3$, with $R_{\text {on}} = G_0^{-1}$, and $G_0 = (13 \, {\mathrm{k}} \Omega )^{-1}$ is the conductance quanta. The evolution of $\lambda (t)$ is described by a polarity-dependent voltage-threshold model^39,63,96,97^:

6 $$\begin{aligned} \dfrac{d \lambda }{dt} = {\left\{ \begin{array}{ll} (|V(t)| - V_{\text {set}}) {\text {sign}}[V(t)], &{} |V(t)| > V_{\text {set}}\\ 0 , &{} V_{\text {reset}}< |V(t)|< V_{\text {set}}\\ b(|V(t)| - V_{\text {reset}}) {\text {sign}}[\lambda (t)], &{} |V(t)| < V_{\text {reset}} \end{array}\right. } \end{aligned}$$

where $V_{\text {set}}$ is the on-threshold and $V_{\text {reset}}$ is the off-threshold, and *b* is a positive constant defining the relative rates of decay of the filament. The following default parameter values are used for all the simulation results presented in this study: $V_{\text {set}} = 10^{-2}$V, $V_{\text {reset}} = 10^{-3}$V and $b = 10$. Experimental validation of this model is presented elsewhere^30,63,97^.

### Centrality

Centrality identifies the most important components in a network, with various definitions used in the literature^74^. In this study, a variation of betweenness centrality is used, based on current flow in circuits proposed by Brandes and Fleischer^68^. The current betweenness centrality of an edge in the network is defined as

7 $$\begin{aligned} c_{EB}(e) = \frac{\sum \limits _{i \ne j \in N}\tau _{ij}(e)}{(N-1)(N-2)}, \end{aligned}$$

where $\tau _{ij}(e)$ is the current flow through edge *e* between nodes *i* and *i* and *N* is the number of nodes in the network.

### Modularity

Modularity is a metric that characterizes the community structure of networks^74–76^. In this study, communities are determined by the Louvain method^98^. The resolution parameter of the detection method is chosen according to a mutual information based method proposed by Ronhovde et al^99^. To capture the influence of network dynamics, we define a modularity weighted by junction conductance:

8 $$\begin{aligned} Q^w = \frac{1}{g} \sum \limits _{i,j} \left[ G(i,j) - \frac{d_i d_j}{g} \right] \delta _{m_i m_j}\qquad , \qquad g = \sum _{i, j \in N} G(i,j) \end{aligned}$$

where $m_i$ is the community that node *i* belongs to, and $\delta$ is the Kronecker delta function.

### Information dynamics

Transfer entropy measures how much information the past of time-series process ${\mathbf {Y}}$ contributes to predicting the next value of time-series process ${\mathbf {X}}$, in the context of the past of ${\mathbf {X}}$. Specifically, transfer entropy is conditional mutual information between these variables, written as^42,48^:

9 $$\begin{aligned} T_{Y \rightarrow X}(k,l)&= I({\mathbf {Y}}_{n}^{(l)};X_{n+1}|{\mathbf {X}}_{n}^{(k)}), \nonumber \\&= \left\langle \log \frac{p(x_{n+1}| {\mathbf {x}}_n^{(k)}, {\mathbf {y}}_n^{(l)})}{p(x_{n+1}|{\mathbf {x}}_n^{(k)})} \right\rangle , \end{aligned}$$

where *n* is a time index for the variables, ${\mathbf {Y}}_{n}^{(l)}$ is the variable representing the past *l* values of the source up to and including time *n*, $X_{n+1}$ the next value of the target at time $n+1$, and ${\mathbf {X}}_{n}^{(k)}$ the past *k* values of the target. The lower case identifiers $(x_{n+1}, {\mathbf {x}}_n^{(k)}, {\mathbf {y}}_n^{(l)})$, indicate a specific realisation of the variables.

Active information storage $A_X$ measures how much information the past of process ${\mathbf {X}}$ contributes to predicting the next value of that process^45,47^. This is a mutual information between ${\mathbf {X}}_{n}^{(k)}$ and $X_{n+1}$:

10 $$\begin{aligned} A_X(k)&= I({\mathbf {X}}_n^{(k)}; X_{n+1}). \end{aligned}$$

In the context of this study, information transfer can be calculated between any two nodes, as the voltage distribution across all the network nodes, and hence junction conductance, changes dynamically in response to the input signal. Transfer entropy (TE) from node *i* to node *j* is calculated based on their respective voltage time-series ${\mathbf {V}}_i$ and ${\mathbf {V}}_j$:

11 $$\begin{aligned} {\mathrm {TE}}_{i,j} = T_{{\mathbf {V}}_i \rightarrow {\mathbf {V}}_j}. \end{aligned}$$

TE activity on the edge connecting nodes *i* and *j* is represented heuristically as a sum of the TEs on both directions of the edge:

12 $$\begin{aligned} {\mathrm {TE}}_{e} = {\mathrm {TE}}_{i,j} + {\mathrm {TE}}_{j,i} \end{aligned}$$

In NWNs, memory is incorporated by the conductance evolution of memristive junctions, whose instantaneous state depends on past history of states. Therefore, active information storage (AIS) is calculated based on the conductance time-series ${\mathbf {G}}$ on junctions, which can be written as:

13 $$\begin{aligned} {\mathrm {AIS}}_e = A_{{\mathbf {G}}}. \end{aligned}$$

TE and AIS are calculated using the JIDT open-source software^46^. Two estimators are employed to capture different aspects of information dynamics. The linear-Gaussian estimator models the variables as having Gaussian distributions with linear interactions; it is fast, and very efficient when the model assumption is correct, but only captures the linear component of an interaction. In contrast, the Kraskov or KSG estimator^100–102^ uses a nearest-neighbour technique to estimate the relationships in a model-free manner; this can detect arbitrary non-linear relationships, though typically requires more data to do so.

### Memory and learning tasks

To investigate the information processing performance of NWNs, two reservoir computing benchmark tasks were implemented on the network: memory capacity and non-linear transformation. For both tasks, the input voltage signal was delivered to one source node and voltage was read out from multiple nodes, replicating experimental implementation^38^.

#### Pre-initialisation

Networks are pre-initialised to different initial states to investigate the effect on task performance. The different initial states are defined as the unique junction conductance distributions captured every 0.05 s, in response to the input Mackey-Glass time-series signal. Then using each stored state as a unique initial state, memory capacity and non-linear transformation tasks are performed and corresponding AIS and TE calculated. Junction conductance levels are reset to zero homogeneously across the network prior to pre-initialisation. However, in physical NWNs it may take on the order of 24 hours for junction conductance levels to reset^30^.

#### Memory capacity

Memory capacity (MC) is a measure of a reservoir’s ability to recall information from its fading memory property^77^. A time series input voltage signal is generated from a uniform random distribution in the interval $[-2,2]$. A linear combination of the network’s state (node voltage) at *t* is applied to reconstruct the previous input at $t - k$. Using $k = 1 ... k_{\mathrm {max}}$ to include different delayed intervals, MC is defined as

14 $$\begin{aligned} {\text {MC}} = \sum \limits _{k=1}^{k_{\mathrm {max}}} {\text {MC}}_k \; , \; {\text{ with }} \; {\text {MC}}_k = \dfrac{\text {cov}^2({\mathbf {u}}_{t-k}, {\mathbf {V}}_t)}{\sigma ^2 ({\mathbf {u}}_{t-k}) \sigma ^2 ({\mathbf {V}}_t)} \; . \end{aligned}$$

where ${\text {MC}}_k$ represents the network’s memory of the input from *k* steps before, $k_{\mathrm {max}} = N$, in which *N* is the number of nodes in the network, ${\mathbf {u}}$ is the input time-series signal and ${\mathbf {V}}$ is the voltage time-series readout from the network.

#### Non-linear transformation

Non-linear waveform transformation tests a reservoir’s learning ability^38^. An input sinusoidal wave is nonlinearly transformed by the network into either a square or sawtooth waveform. Similar to the MC test, linear combinations of the network’s states are used to fit the target signal:

15 $$\begin{aligned} {\mathbf {Y}} = \mathbf \omega \cdot {\mathbf {V}}, \end{aligned}$$

where $\mathbf \omega$ is the fitted weight trained by linear regression and ${\mathbf {V}}$ is the voltage readout from the network. The accuracy is calculated as $1 - {\text {RNMSE}}$, where

16 $$\begin{aligned} {\text {RNMSE}} = \sqrt{\dfrac{\sum ({\mathbf {Y}} - {\mathbf {T}})^2}{\sum {\mathbf {T}}^2}}. \end{aligned}$$

is the root-normalised mean square error, and where ${\mathbf {Y}}$ is the trained result while ${\mathbf {T}}$ represents the target time-series signal.

## Supplementary Information

Supplementary Information.
