# Supplementary material for: Information dynamics in neuromorphic nanowire networks
Source: Sci Rep. 2021 Jun 22;11:13047. doi: 10.1038/s41598-021-92170-7 (PMC8219687; doi:10.1038/s41598-021-92170-7)
Supplement: Supplementary file 1 — Supplementary Information. [file 41598_2021_92170_MOESM1_ESM.pdf]

Supplementary Information: Information dynamics in neuromorphic nanowire networks

Ruomin Zhu, Joel Hochstetter, Alon Loeffler, Adrian Diaz-Alvarez, Tomonobu Nakayama, Joseph T. Lizier, Zdenka Kuncic

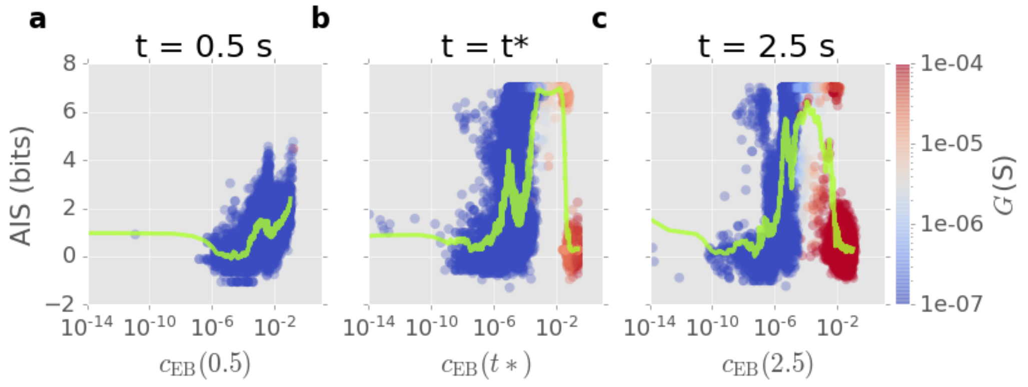

Figure 1: Junction active information storage (AIS) as a function of edge betweeness centrality c_{EB} calculated for 50 simulations of a 100-node network with varying source-drain locations (fixed distance) at three different time points: (a) $t = 0.5$ s; (b) $t = t^*$, where $t^* = 1.22$ s is network activation time; and (c) $t = 2.5$ s. Each data point represents one junction at the specified time. The colourbar represents the corresponding junction conductance. The yellow curve is a moving window average (size 0.1 s) of the scatter points calculated by sorting the scatter points based on their c_{EB} values and averaging c_{EB}, AIS values within each window.

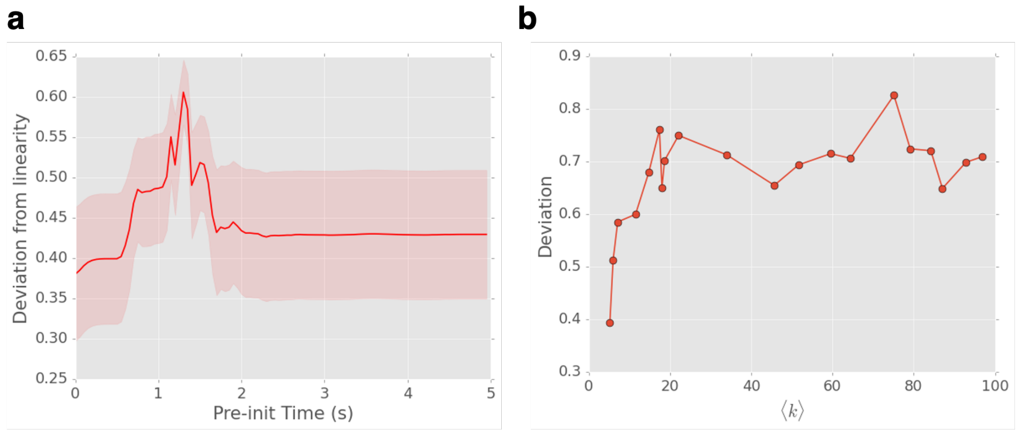

Figure 2: Deviation from linearity of NWNs. (a) Deviation from linearity as a function of pre-initialisation time for a 100 nanowire/ 261 junction NWN realisation. (b) Deviation from linearty of NWNs as a function of mean connectivity $\langle k \rangle$.

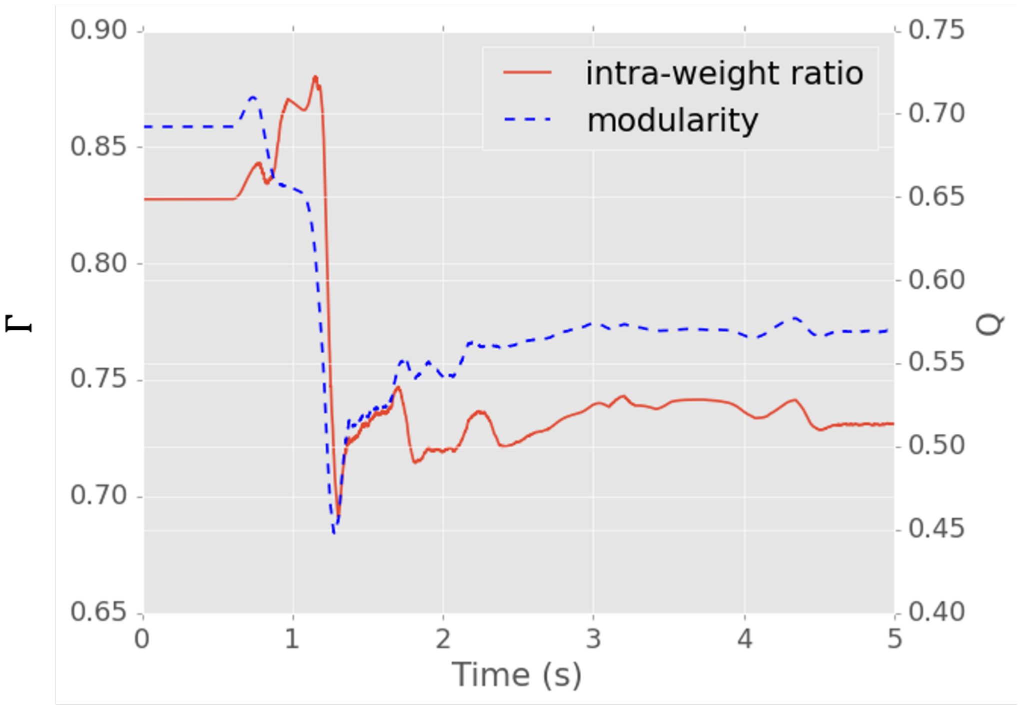

Figure 3: Intra-module weight ratio Γ and modularity Q as a function of time. Red curve is the time-series of the sum of weights on intra-module edges normalized by the total weights in the network ($\Gamma = \frac{\sum_{m_i=m_j, i,j \in N} W_{i,j}}{\sum_{i,j \in N} W_{i,j}}$). Dashed blue curve represents the modularity time-series.

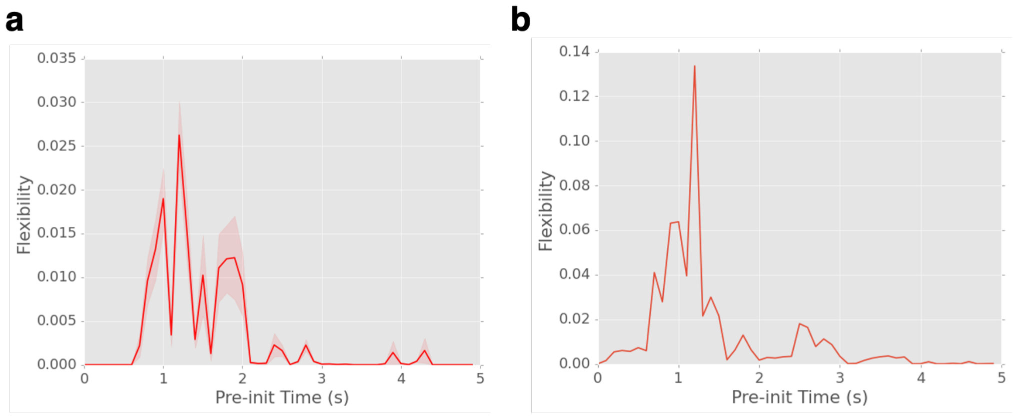

Figure 4: Flexibility of NWNs during learning tasks. (a) Flexibility of NWN as a function of pre-initialisation time during the memory capacity (MC) task. (b) Flexibility of NWN as a function of pre-initialisation time during the non-linear transformation (NLT) task.
